# Accounting for Subgroup Structure in Line-Transect Abundance Estimates of False Killer Whales (*Pseudorca crassidens*) in Hawaiian Waters

**DOI:** 10.1371/journal.pone.0090464

**Published:** 2014-02-28

**Authors:** Amanda L. Bradford, Karin A. Forney, Erin M. Oleson, Jay Barlow

**Affiliations:** 1 Protected Species Division, Pacific Islands Fisheries Science Center, National Marine Fisheries Service, National Oceanic and Atmospheric Administration, Honolulu, Hawaii, United States of America; 2 Marine Mammal and Turtle Division, Southwest Fisheries Science Center, National Marine Fisheries Service, National Oceanic and Atmospheric Administration, Santa Cruz, California, United States of America; 3 Marine Mammal and Turtle Division, Southwest Fisheries Science Center, National Marine Fisheries Service, National Oceanic and Atmospheric Administration, La Jolla, California, United States of America; Institut Pluridisciplinaire Hubert Curien, France

## Abstract

For biological populations that form aggregations (or clusters) of individuals, cluster size is an important parameter in line-transect abundance estimation and should be accurately measured. Cluster size in cetaceans has traditionally been represented as the total number of individuals in a group, but group size may be underestimated if group members are spatially diffuse. Groups of false killer whales (*Pseudorca crassidens*) can comprise numerous subgroups that are dispersed over tens of kilometers, leading to a spatial mismatch between a detected group and the theoretical framework of line-transect analysis. Three stocks of false killer whales are found within the U.S. Exclusive Economic Zone of the Hawaiian Islands (Hawaiian EEZ): an insular main Hawaiian Islands stock, a pelagic stock, and a Northwestern Hawaiian Islands (NWHI) stock. A ship-based line-transect survey of the Hawaiian EEZ was conducted in the summer and fall of 2010, resulting in six systematic-effort visual sightings of pelagic (*n* = 5) and NWHI (*n* = 1) false killer whale groups. The maximum number and spatial extent of subgroups per sighting was 18 subgroups and 35 km, respectively. These sightings were combined with data from similar previous surveys and analyzed within the conventional line-transect estimation framework. The detection function, mean cluster size, and encounter rate were estimated separately to appropriately incorporate data collected using different methods. Unlike previous line-transect analyses of cetaceans, subgroups were treated as the analytical cluster instead of groups because subgroups better conform to the specifications of line-transect theory. Bootstrap values (*n* = 5,000) of the line-transect parameters were randomly combined to estimate the variance of stock-specific abundance estimates. Hawai’i pelagic and NWHI false killer whales were estimated to number 1,552 (CV = 0.66; 95% CI = 479–5,030) and 552 (CV = 1.09; 95% CI = 97–3,123) individuals, respectively. Subgroup structure is an important factor to consider in line-transect analyses of false killer whales and other species with complex grouping patterns.

## Introduction

Line-transect methods are commonly used to estimate the density and abundance of biological populations and have been widely applied to cetaceans [1], [2], [3], [4], [5]. When individuals in a study population occur in aggregations (or clusters) of individuals, the cluster size (i.e., number of individuals) of a detection becomes an integral component of line-transect abundance estimation and should be accurately measured [6]. Cluster size in cetaceans has traditionally been interpreted as the total size of a detected group [7], but visual observation may underestimate group size if group members are spatially diffuse or prolonged divers. The potential to underestimate group size is particularly pronounced for cetacean groups that exhibit subgroup structure [8]. For example, groups of false killer whales (*Pseudorca crassidens*) can consist of multiple dispersed subgroups, and total group size may be underestimated if encounter duration is insufficient [9]. Further, these subgroups can be separated by large enough distances that a spatial mismatch is created between an observed group and the theoretical framework of line-transect analysis, which treats clusters as if they occupy a single point (i.e., the center of the cluster) in two-dimensional space.

False killer whales are widely distributed in tropical and warm-temperate waters, although they are infrequently encountered at sea [9], [10]. Much of what is known about the ecology and social structure of this species comes from a longitudinal study of a small, island-associated population in the main Hawaiian Islands [9], [11]. This population has experienced a precipitous decline in recent decades, was estimated to number 151 (CV = 0.20) individuals from 2006 to 2009, and was determined to be a distinct population segment (DPS) under the U.S. Endangered Species Act (ESA) [12]. In 2012, the U.S. National Marine Fisheries Service (NMFS) listed the main Hawaiian Island false killer whale DPS as endangered under the ESA (77 FR 70915, 28 November 2012). The main Hawaiian Islands false killer whale population is one of two management stocks within the U.S. Exclusive Economic Zone of the Hawaiian Archipelago (Hawaiian EEZ) that have been recognized by NMFS since 2008 [13]. The insular main Hawaiian stock has been differentiated from a more broadly distributed pelagic stock using genetic, photo-identification, and movement data [9], [11], [14], [15].

False killer whales are known to depredate catch in the Hawai’i-based pelagic longline fisheries. This depredation results in economic losses to the fisheries and creates the potential for false killer whale mortality or serious injury [16]. Assessments mandated by the U.S. Marine Mammal Protection Act (MMPA) have shown that the bycatch of pelagic false killer whales in the Hawaiian EEZ exceeds allowable levels [13]. Accordingly, a Take Reduction Team was convened by NMFS in 2010, which resulted in a Take Reduction Plan for reducing false killer whale bycatch (77 FR 71260, 29 November, 2012). Abundance estimates are used in the MMPA stock assessment process to estimate sustainable levels of bycatch [17]. The abundance of the pelagic false killer whale stock was estimated to be 484 (CV = 0.93) individuals based on data collected during the first Hawaiian Cetacean Ecosystem Assessment Survey (HICEAS), a line-transect survey of the Hawaiian EEZ in 2002 [18]. However, abundance estimates associated with marine mammal stock assessments are considered outdated after eight years [17]. Thus, an update to the 2002 estimate of pelagic false killer abundance is needed.

A second HICEAS was conducted in 2010 as a collaborative effort between the NMFS Pacific Islands Fisheries Science Center (PIFSC) and the Southwest Fisheries Science Center (SWFSC). As with the initial HICEAS in 2002, the primary objective of HICEAS 2010 was to carry out line-transect surveys within the Hawaiian EEZ to estimate the abundance of cetaceans, including the pelagic stock of false killer whales. Genetic, photo-identification, and satellite tagging data were also collected during HICEAS 2010. A comparison of these data to those from previous surveys indicated that false killer whales within the insular waters of the Northwestern Hawaiian Islands (NWHI) warrant recognition as a separate and third stock, distinct from both the main Hawaiian Islands and pelagic stocks [19], [20]. Available evidence suggests that the NWHI stock is island-associated like that of the main Hawaiian Islands, although the mechanisms driving the differentiation between the two populations are unclear [20]. The objective of this paper is to estimate the abundance of false killer whales in the pelagic region of the Hawaiian EEZ and the insular waters of the NWHI using the line-transect sightings from HICEAS 2010, while specifically accounting for the complex grouping patterns of false killer whales. These abundance estimates are important for the assessment and management of pelagic and NWHI false killer whales, and the associated analytical considerations are applicable to other biological populations with subgroup structure.

## Materials and Methods

### Ethics Statement

HICEAS 2010 was conducted under MMPA permit 14097 issued to the SWFSC. Survey effort within the Papahānaumokuākea Marine National Monument was conducted under permit PMNM-2010-053 issued to JB and EMO. At the time of the survey, Institutional Animal Care and Use Committee (IACUC) approval was not required for NMFS permits, but current NMFS permits allowing similar methods as employed during HICEAS 2010 have been IACUC approved.

### Data Collection

**Figure 1:**
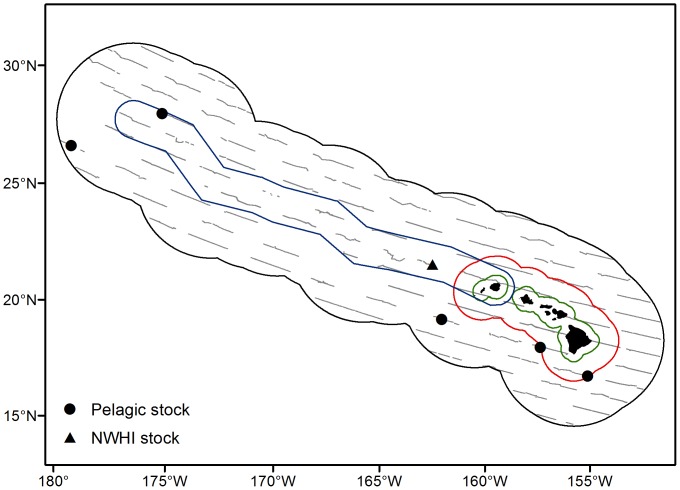
False killer whale sightings in the Hawaiian EEZ. Systematic-effort sightings of false killer whales from the Hawai’i pelagic and Northwestern Hawaiian Islands (NWHI) stocks made during HICEAS 2010. The black outline represents the Hawaiian EEZ. The colored outlines indicates the area used exclusively by the insular main Hawaiian Islands stock (green), the overlap zone between the main Hawaiian Islands and pelagic stocks (red), and the boundary of the NWHI stock (blue). The fine gray lines depict systematic-effort survey coverage in Beaufort sea states from 0 to 6.

The second HICEAS was conducted during the summer–fall of 2010 aboard two National Oceanic and Atmospheric Administration (NOAA) research vessels. Survey dates for the 68-m NOAA Ship *McArthur II* (McII) were 4 August to 10 December 2010, and survey dates for the 68-m NOAA Ship *Oscar Elton Sette* (OES) were 1 September to 29 October 2010. The HICEAS 2010 study area was the U.S. EEZ of the Hawaiian Archipelago (Fig. 1). The line-transect survey design was similar to that of HICEAS 2002 [21], with parallel transect lines that were oriented WNW–ESE to minimize the effects of the dominant swells in the region. These transect lines formed a grid that provided comprehensive coverage of the study area. The original 2002 grid was established by randomly placing an initial transect line and then positioning other transect lines parallel at a spacing of 85 km apart. Transect lines in 2010 were placed midway between each of the 2002 lines, allowing for denser coverage of the study area over the two surveys. Unlike HICEAS 2002, HICEAS 2010 survey effort was not stratified to intensively sample the main Hawaiian Islands. Thus, transect density was roughly uniform throughout the study area. Both vessels surveyed at a speed of 10 kts. Transits to and from ports and island circumnavigations were not a part of the systematic survey grid, although the visual observers generally remained on-effort, following standard observation protocols. Sightings made during this nonsystematic effort and while off-effort were not suitable for the estimation of false killer whale encounter rates in the study area, but were used to inform other line-transect parameters, as appropriate.

The general visual observation methods employed during HICEAS 2010 are well established, having been in use by the SWFSC for the last three decades [22], including surveys in the Pacific Islands region [18], [21]. Observation teams comprised six observers who rotated through three viewing positions and searched for cetaceans using 25× binoculars and unaided eyes from the approximately 15-m flying bridge. If cetaceans were sighted within 5.6 km of the trackline by an on-effort observer, the ship diverted from the trackline toward the sighting so that species, proportion of species present (for mixed-species groups), and an estimate of group size could be determined. A protocol specific to sightings of false killer whales (hereafter referred to as the group-size estimation protocol) was established for HICEAS 2010 to obtain more accurate estimates of group size (see below). Along with basic environmental data (e.g., Beaufort sea state, swell height, and visibility), data recorded for each sighting included the time, location, species, initial bearing and radial distance to the sighting (used to compute the perpendicular distance from the trackline), identity of observers and their independent estimates of group size (“best,” “high,” and “low”), and proportion of each species present. If weather, animal behavior, and research priorities permitted, a small boat was launched following species identification and enumeration to collect photo-identification images and biopsy samples for the purposes of individual and stock identification. In some cases, satellite tags were also deployed to track individual movements and inform stock boundaries. The genetic, photo-identification, and tagging data were used to assign each false killer whales sighting to either the pelagic, NWHI, or main Hawaiian Islands stock [9], [11], [14], [15], .

### Group-Size Estimation Protocol

As introduced, false killer whale groups can comprise several spread out subgroups, creating the potential to underestimate total group size [9]. A line-transect survey of the U.S. EEZ around Palmyra demonstrated that some false killer whale subgroups detected acoustically were missed by the visual observers [18]. The group-size estimation protocol implemented during HICEAS 2010 combined visual and acoustic information to find and enumerate the sizes of subgroups, allowing for a more accurate estimation of total group size. Specifically, when false killer whales were visually detected, acoustic information was used to direct the ship to the perceived center of the group. Minor and infrequent turns were made only as needed to improve the acoustic localization and sighting distance of subgroups. The on-effort visual observers, supplemented by off-effort observers, were responsible for estimating the sizes of subgroups detected by either observation method, with one observer assigned to each subgroup to ensure complete coverage. Passage through the group continued until no further subgroups were acoustically or visually detected ahead of the beam of the ship.

**Figure 2:**
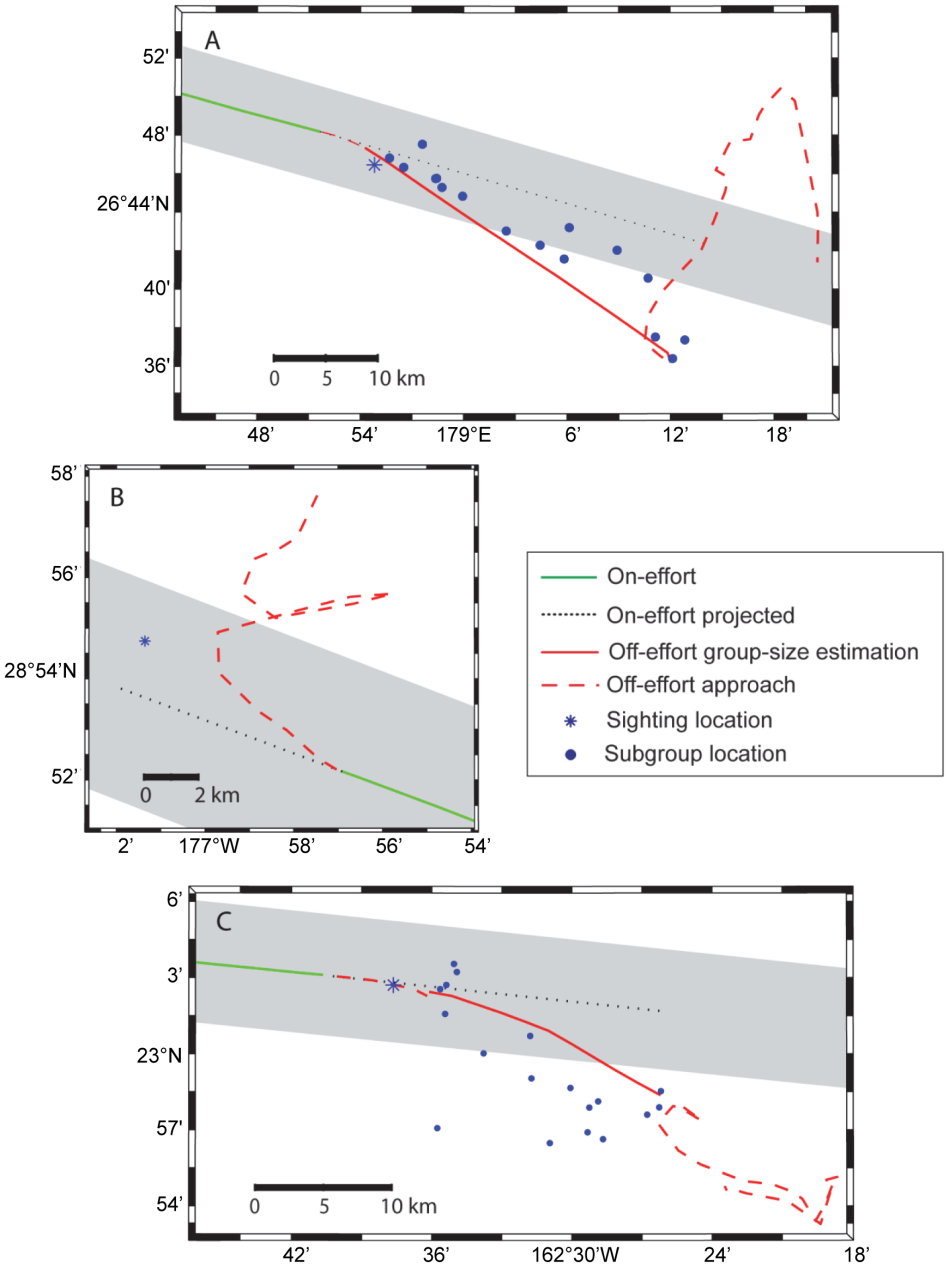
False killer whale group size estimation protocol. Schematics of the systematic-effort false killer whales sightings for which the group-size estimation protocol was attempted: *McArthur II* (McII) sightings 241 (A) and 35 (B) for the pelagic stock and *Oscar Elton Sette* sighting 86 (C) for the Northwestern Hawaiian Islands stock. “On-effort projected” is the track the ship would have taken if systematic-effort status had been maintained. “Off-effort group-size estimation” represents the implementation of the group-size estimation protocol, with the localized subgroups shown as blue circles (except for McII sighting 35, as the protocol was not successfully executed). “Off-effort approach” is the track associated with approaching the group for photo-identification and biopsy sampling (individual subgroups were no longer localized). The sighting location refers to the original visual detection that prompted the group-size estimation protocol. The gray shading denotes the 4.5 km analytical truncation distance.

In practice, the group-size estimation protocol was difficult to implement due to logistical constraints, technical difficulties, and whale behavior. Therefore, it was not successfully executed for all false killer whale sightings during HICEAS 2010. Of the six systematic-effort sightings (Table 1), the group size estimation protocol was attempted for three, although the execution was unsuccessful in one case (i.e., the number, sizes, and locations of subgroups could not be determined) (Fig. 2). In addition to the protocol challenges, considering false killer whale groups in their entirety resulted in unanticipated challenges when attempting to apply the rules for clustered objects dictated by line-transect theory. Specifically, a detected group (or cluster) should only be considered a sighting for analysis if the center of the group is within the analytical truncation distance [6]. As reported below, the span of false killer whale groups can exceed the transect strip width, as well as the visual sighting horizon, creating a spatial mismatch between a sighted group and the line-transect theoretical framework. Incorporating all subgroups into an estimate of group size would overestimate density in the study area due to the inclusion of individuals outside the truncation distance. Subjecting false killer whale detections to the group center criteria is impractical because: 1) the center of a large, dispersed, and mobile group can be difficult to identify; 2) the group center does not have the intended relevance to the detection process [6], and 3) the criteria could lead to the loss of sightings of this rarely encountered species.

**Table 1:** Details of HICEAS 2010 false killer whale sightings made during systematic (S) and nonsystematic (N) survey effort and while off-effort (O).

Date	Ship	Sighting	Effort type	Informed parameter	Group size	Stock	Assignment basis
09/01/10	McII	35	S	*f*(0), *n_i_*/*L_i_*	22.6	Pelagic	G, P
09/05/10	McII	47	O		10.3	Pelagic	P, L
09/07/10	McII	61	O	*E*(*s*)	29.9	Pelagic	P, L
09/10/10	McII	74	O		18.3	Pelagic	G, P
09/26/10	OES	86	S	*f*(0), *E*(*s*), *n_i_*/*L_i_*	52.0	NWHI	G, P
09/27/10	McII	98	S	*f*(0), *n_i_*/*L_i_*	1.9	Pelagic	G, P
09/28/10	McII	103	S	*f*(0), *n_i_*/*L_i_*	1.0	Pelagic	G, P
10/07/10	McII	140	N	*f*(0)	12.1	NWHI	G, P
10/07/10	McII	142	N	*f*(0)	1.7	NWHI	X
10/20/10	McII	200	O		8.8	NWHI	G, P, T
10/21/10	McII	206	N		20.4	NWHI	G, P, T
10/29/10	McII	224	N	*f*(0)	1.0	MHI	L
10/31/10	McII	231	S	*f*(0), *n_i_*/*L_i_*	1.0	Pelagic	L
11/10/10	McII	241	S	*f*(0), *E*(*s*), *n_i_*/*L_i_*	41.0	Pelagic	G, P

Sightings were used, as appropriate, to inform estimation of the line-transect parameters, where *f*(0) relates to the detection function, *E*(*s*) is the mean cluster size, and *n_i_*/*L_i_* is the stock-specific encounter rate.

Group size is either the geometric mean of the best estimates of the observers or the sum of the best estimates of subgroup size.

Sightings were assigned to one of three stocks: the pelagic, Northwestern Hawaiian Islands (NWHI), or main Hawaiian Islands (MHI) based on genetics (G), location (L), photo-identification (P), proximity (X), or tagging (T).

These post-hoc considerations led to the determination that false killer whale subgroups, not groups, more appropriately represent a detectable unit and better conform to the line-transect definition of a cluster [6]. Further, line-transect methods are robust to the assumption that detections are independent, so quantifying the overarching group arrangement is not required [23]. As such, in contrast to other cetacean line-transect analyses that are based on detected groups, subgroups served as the analytical unit in the present abundance estimation. However, this approach required careful treatment (detailed below) of the sightings made using the group-size estimation protocol since the protocol was designed for the group-based analytical framework and was not always successfully executed.

### Abundance Estimation

Multiple-covariate line-transect methods are increasingly being used to estimate the density and abundance of cetaceans [22] and were previously applied to the Hawai’i pelagic stock of false killer whales [18], [21]. The multiple-covariate framework accounts for heterogeneity from covariates other than perpendicular distance in the detection function [24], which can be modeled using sightings pooled from multiple surveys, although the remaining parameters are estimated using only sightings from the focal survey [21]. The ability to model detection probabilities from an enlarged pool of sightings is advantageous for study regions such as the Hawaiian EEZ, where cetacean sighting rates are comparatively low [21]. However, the estimation of the other parameters by a restricted set of sightings limits the uncertainty that can be represented by those parameters, particularly when sightings are few in number. Further, a preliminary evaluation of the multiple-covariate approach found that it would not sufficiently accommodate the variability introduced by the group-size estimation protocol during HICEAS 2010. Therefore, to estimate the density of false killer whales in the pelagic region of the Hawaiian EEZ and the insular waters of the NWHI, the conventional form of the line-transect estimator was employed:
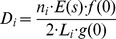
(1)where *D_i_* is the density of stock *i*, *n_i_* is the number of systematic-effort subgroup detections of stock *i*, *E*(*s*) is the expected size (number of individuals) of false killer whale subgroups, *f*(0) is the probability density function of the perpendicular detection distances evaluated at zero distance, *L_i_* is the length of systematic transect lines accomplished in the portion of the study area associated with each stock *i*, and *g*(0) is the probability of detection on the trackline. Data from other sightings and surveys were incorporated into the estimation of some parameters because of the limited number of HICEAS 2010 systematic-effort sightings. Also, the varying implementation of the group-size estimation protocol meant that not all of the systematic-effort sightings had the data needed to estimate each of the parameters. For these reasons, the parameters were estimated separately, as described below, using appropriate data that met parameter-specific assumptions. Variances for all parameters were obtained using bootstrap methods.

### Detection Function

To achieve a more robust sample size for modeling the detection function of subgroups, sightings of false killer whales from HICEAS 2010 were pooled with a subset of false killer whale sightings made during SWFSC and PIFSC line-transect surveys of the eastern tropical and central North Pacific between 1986 and 2009. This combined data set includes a small number of sightings that were collected during nonsystematic survey effort. For all of the sightings, the initial detection was assumed to represent a single subgroup that may or may not have been a part of a larger group. Thus, despite the different approaches used to estimate total group size once a sighting was detected, the HICEAS 2010 and earlier sightings were regarded as comparable in terms of the detection process.

Potential heterogeneity introduced by pooling sightings from different surveys was minimized by restricting the sample to sightings collected under conditions similar to those encountered during HICEAS 2010. Because there were no mixed-species sightings of false killer whales during HICEAS 2010, multispecies sightings from previous years were excluded from pooling to eliminate the influence of other species on the detection process. An exploratory multiple-covariate analysis was conducted to identify additional sources of heterogeneity. Specifically, a half-normal model was used to evaluate the detection probabilities of available sightings as a function of perpendicular distance from the trackline and relevant covariates [7]. Only half-normal models were employed in modeling the detection function because they exhibit greater stability than other models when fitting cetacean sighting data [25]. Geographical region (eastern tropical or central North Pacific) and Beaufort sea state (restricted to values between 0 and 6) were identified as important determinants of detection probability. These two covariates are likely correlated because calm conditions (Beaufort states 0–2) are not commonly encountered within the central North Pacific. Thus, to make the pooled sample reflect the higher sea state conditions characteristic of the central North Pacific, only sightings made in Beaufort states 3–6 were included. A two-sample Kolmogorov-Smirnov test was performed on the pooled sightings to evaluate the similarity in the distribution of Beaufort states by region. A lack of significant difference between these distributions was used to indicate that the detected heterogeneity had been minimized.

A half-normal model (with no adjustments) was fitted to the perpendicular distances of the combined data set, which was truncated at 4.5 km to improve model fit [6], [18], [21]. The program Distance [26] was used to estimate *f*(0) and its inverse, the effective strip width (*ESW*; the distance from the trackline for which as many individuals were detected beyond as were missed within) and to obtain a bootstrap estimate (*n* = 5,000 iterations) of the coefficient of variation (CV).

### Expected Subgroup Size

Subgroup structure was not explicitly detailed or quantified in false killer whale sightings from earlier line-transect surveys. Thus, there are few existing observations of subgroup size. The only available values are those resulting from the group-size estimation protocol during HICEAS 2010, as well as a few observations made using a new subgroup-oriented passing mode protocol introduced during a 2011 survey of the U.S. EEZ around Palmyra Atoll (PIFSC, unpublished data). These observations of subgroup size were averaged to estimate *E*(*s*). Although infrequent, when more than one observer provided a “best” estimate of size for a given subgroup, the geometric mean of the estimates was used [21]. In some cases, observers recorded a “high” and “low” value of subgroup size, but were unable to provide a “best” estimate. For these subgroups, an average “best”:“low” ratio, computed from subgroups with the full complement of estimates, was used to determine a “best” estimate of subgroup size. The pooled values of subgroup size were randomly sampled with replacement 5,000 times to estimate the CV of *E*(*s*).

### Encounter Rate

Encounter rates in cetacean surveys are typically based on the number of sightings per unit of effort distance [21]. In the present analysis, the encounter rate of each stock (*n_i_*/*L_i_*) represents the number of subgroup detections divided by the length of transect lines surveyed. Counting sightings for which the group-size estimation protocol was attempted as a single detection would underestimate *n_i_*/*L_i_* (and thus density) because these sightings contained multiple subgroups. However, because the group-size estimation protocol directed the ship away from the trackline and toward subgroups, it is unknown how many of the subgroups would have been visually detected had the ship remained on the trackline. Therefore, the expected number of detected subgroups for these sightings was determined probabilistically.

Estimating the expected number of visually detected subgroups for the group-size estimation protocol sightings first involved projecting an on-effort trackline representing the path the ship would have taken past the group if it had remained in passing mode on the initial detection (Fig. 2). This projected on-effort trackline continued until all identified subgroups would have passed the beam of the ship. The initial location of each subgroup was determined from the recorded bearing and distance of the subgroup from the actual path of the ship. Perpendicular distances from these locations to the projected on-effort trackline were then calculated. Subgroups more than 4.5 km from the projected trackline were not considered further, as they were beyond the analytical truncation distance. Of the subgroups within 4.5 km of the projected trackline, the subgroup closest to the location of the initial visual detection was considered to represent the initial visual detection and assigned a detection probability of 100%. Detection probabilities for the remaining subgroups were based on the distance of the subgroup from the projected trackline and the estimated detection function. These probabilities were summed to compute the expected number of subgroups that would have been detected if the vessel had remained in passing mode.

For the sighting in which the group-size estimation protocol was unsuccessfully executed, the number and locations of subgroups were unknown (Fig. 2B). Therefore, the number of subgroups and their average detection probabilities were estimated from other available data. The total group size of the sighting was divided by the point estimate of *E*(*s*) to estimate the number of subgroups present. To determine how many of these subgroups were within the 4.5 km truncation distance, the estimated number of subgroups was multiplied by the average proportion of subgroups within 4.5 km of the projected trackline for the sightings in which the group-size estimation protocol was successfully implemented. The first of the remaining subgroups was assigned a detection probability of 100%, while the others were assigned the average detection probability estimated for the study (i.e., *ESW* divided by the truncation distance). The effort distance associated with all sightings in which the group-size estimation protocol was attempted was adjusted to include the length of the projected on-effort trackline.

The expected number of detected subgroups for each sighting was summed over all stock-specific sightings to produce *n_i_*. The variance of *n_i_*/*L_i_* was estimated using a bootstrap procedure. Specifically, the systematic survey coverage of the range of each stock was divided into 150 km effort segments, which approximates the distance surveyed in a single day [21]. These effort segments and their associated sightings were randomly sampled with replacement 5,000 times. When a segment was drawn that contained a group-size estimation protocol sighting, the number of detected subgroups was stochastically determined within the bootstrap based on the estimated detection probabilities described above. For the sighting in which the group-size estimation protocol was unsuccessfully executed, uncertainty in the number of subgroups present was included in the bootstrap by drawing a random sample of subgroups from the available observations used to estimate *E*(*s*) until the sum of all subgroups in a draw totaled the estimate of total group size recorded for the sighting.

### Density and Abundance

Based on the range of subgroup sizes observed in the present study, the *g*(0) estimate (0.76, CV = 0.14) for small groups (<20 individuals) of delphinids [2] was employed in the analysis. Bootstrap values (*n* = 5,000) were obtained by modeling *g*(0) as a logit-transformed deviate with a mean and variance chosen to give the estimated *g*(0) and CV [21]. For each stock, density (individuals per km^2^) was calculated using Equation (1) and the point estimate of each parameter. Variance in density was estimated by randomly combining the 5,000 bootstrap values of *f*(0), *E*(*s*), *n_i_*/*L_i_*, and *g*(0). Abundance was determined by multiplying the density values by the portion of the study area associated with each false killer whale stock (Fig. 1). For pelagic false killer whales, this area is 2,378,724 km^2^, which encompasses the Hawaiian EEZ minus the land masses of the main and Northwestern Hawaiian Islands, as well as waters within 40 km of the main Hawaiian Islands, which are considered to be occupied exclusively by the main Hawaiian Islands stock of false killer whales [12]. The full range of the recently documented NHWI stock of false killer whales is unknown, but a boundary combining the Papahānaumokuākea Marine National Monument with insular waters of the westernmost main Hawaiian Islands (Kaua’i and Ni’ihau) is most consistent with available photo-identification and satellite tagging data [20]. The area incorporated is 414,743 km^2^, which includes the Monument with its eastern edge extended to a 93 km buffer east of Kaua’i minus the land mass of the NWHI, Kaua’i, and Ni’ihau. Lognormal 95% confidence intervals (CI) [27] were computed for each abundance estimate.

## Results

### Survey Sightings

On-effort visual searches for pelagic false killer whales in the Hawaiian EEZ encompassed 15,617 km of systematic transects in Beaufort sea states from 0 to 6 (Fig. 1), with most (94.4%) of the effort conducted in Beaufort states 3–6. Five systematic-effort sightings of pelagic stock false killer whales were made during HICEAS 2010 (Table 1). The group-size estimation protocol was successfully implemented for one systematic-effort pelagic sighting (McII 241), which included 16 localized subgroups that spanned over 35 km and were tracked for more than 2 hours (Fig. 2A). The group-size estimation protocol was attempted for another systematic-effort pelagic sighting (McII 35), but the execution was unsuccessful and the observed subgroups were not quantified or localized (Fig. 2B). The three remaining systematic-effort sightings consisted of single, small subgroups (Table 1). The acoustic observers were off-effort during these sightings, precluding the use of the group-size estimation protocol, but no additional subgroups were visually detected.

On-effort visual searches for false killer whales within the assumed stock range of the NWHI stock covered 2,901 km of systematic transects in Beaufort sea states from 0 to 6, with most (86.0%) of the effort also conducted in Beaufort states 3–6 (Fig. 1). Only one systematic-effort sighting was made of false killer whales from the NWHI stock (Table 1). The group-size estimation protocol was successfully implemented for this sighting (OES 86), which included 18 subgroups that spanned over 25 km and were also tracked for more than 2 hours (Fig. 2C). Eight additional nonsystematic-effort (n = 4) and off-effort (n = 4) sightings of false killer whales were made during HICEAS 2010, including three sightings from the pelagic stock, four from the NWHI stock, and one from the main Hawaiian Islands stock (Table 1). These sightings were not part of the systematic visual line-transect survey and were therefore excluded from the encounter rate estimation. However, three sightings (McII 140, 142, and 224) collected during nonsystematic effort were suitable for inclusion in the pooled sample that was used to estimate the detection function, and one off-effort sighting (McII 61) conducted using the group-size estimation protocol provided estimates of subgroup size (Table 1).

### Line-Transect Parameters

**Figure 3:**
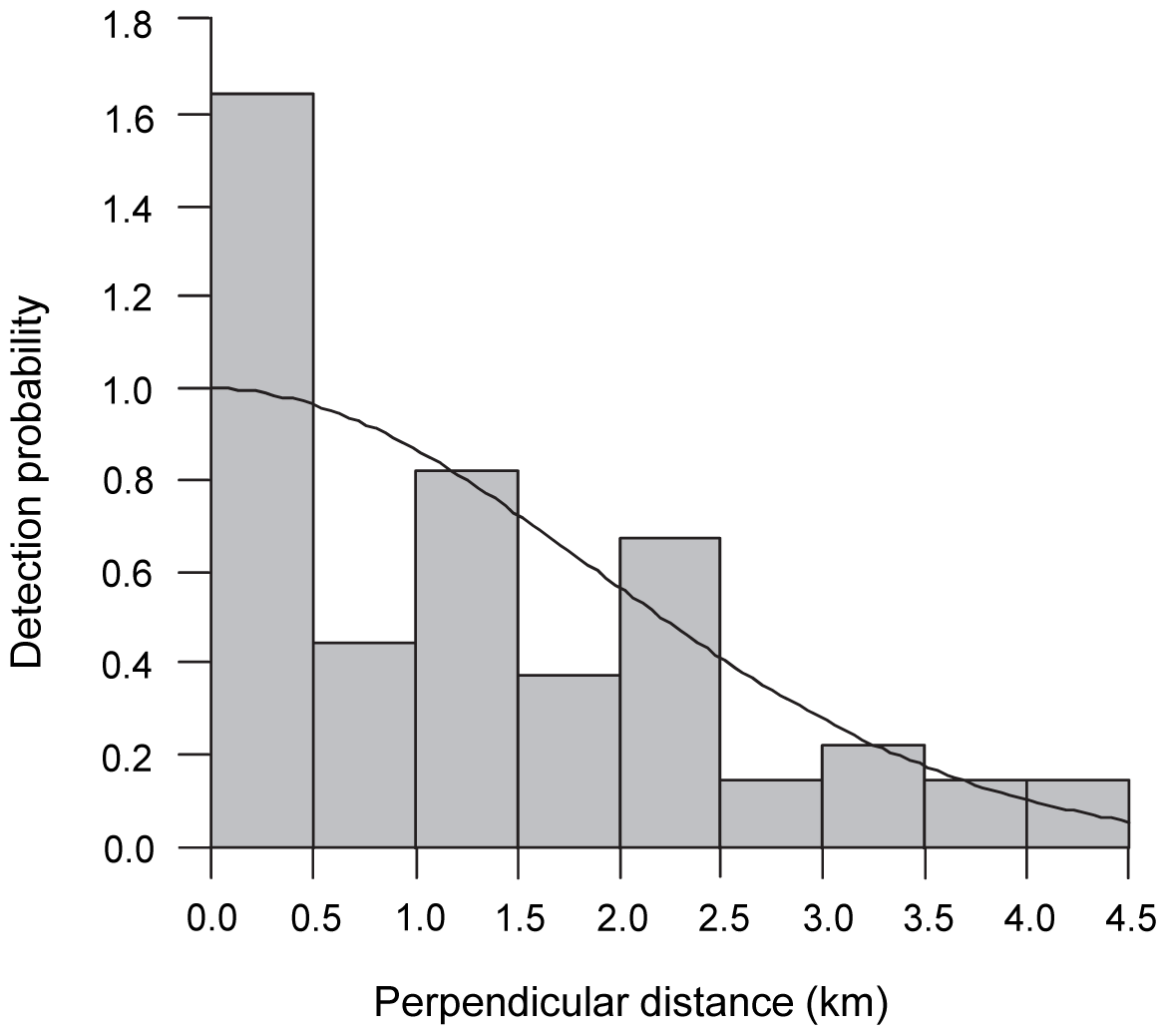
False killer whale detection function. Histogram of false killer whale sightings (n = 62) by perpendicular distance from the trackline and fit of the half-normal model used to estimate the detection function of subgroups.

A total of 62 systematic-effort (*n* = 57) and nonsystematic-effort (*n* = 5) false killer whale sightings made from 1986 to 2010 met the pooling criteria for modeling subgroup detection probabilities. Thirty-nine of the sightings (all systematic-effort) are from the eastern tropical Pacific, while 23 sightings (including the six systematic-effort and three applicable nonsystematic-effort HICEAS 2010 sightings) are from the central North Pacific (Table 2). The two-sample Kolmogorov-Smirnov test did not detect a difference in the distribution of Beaufort sea states by region (*D* = 0.13, *p*-value = 0.97), indicating that the heterogeneity associated with Beaufort state and region had been minimized. The pooled sample includes sightings that occurred disproportionately more often in the distance bin closest to the trackline (Fig. 3). The resulting detection function (Fig. 3) and bootstrap resampling led to an *f*(0) estimate of 0.43 (CV = 0.11) km^−1^ (*ESW* = 2.31 km).

**Table 2:** Distribution of false killer whale sightings made in the eastern tropical Pacific (ETP) and central North Pacific (CNP) from 1986 to 2010 according to Beaufort sea state.

Beaufort	ETP	CNP
3	12	9
4	17	6
5	10	5
6	0	3

Perpendicular trackline distances associated with these sightings were used to model the detection function of false killer whale subgroups.

**Figure 4:**
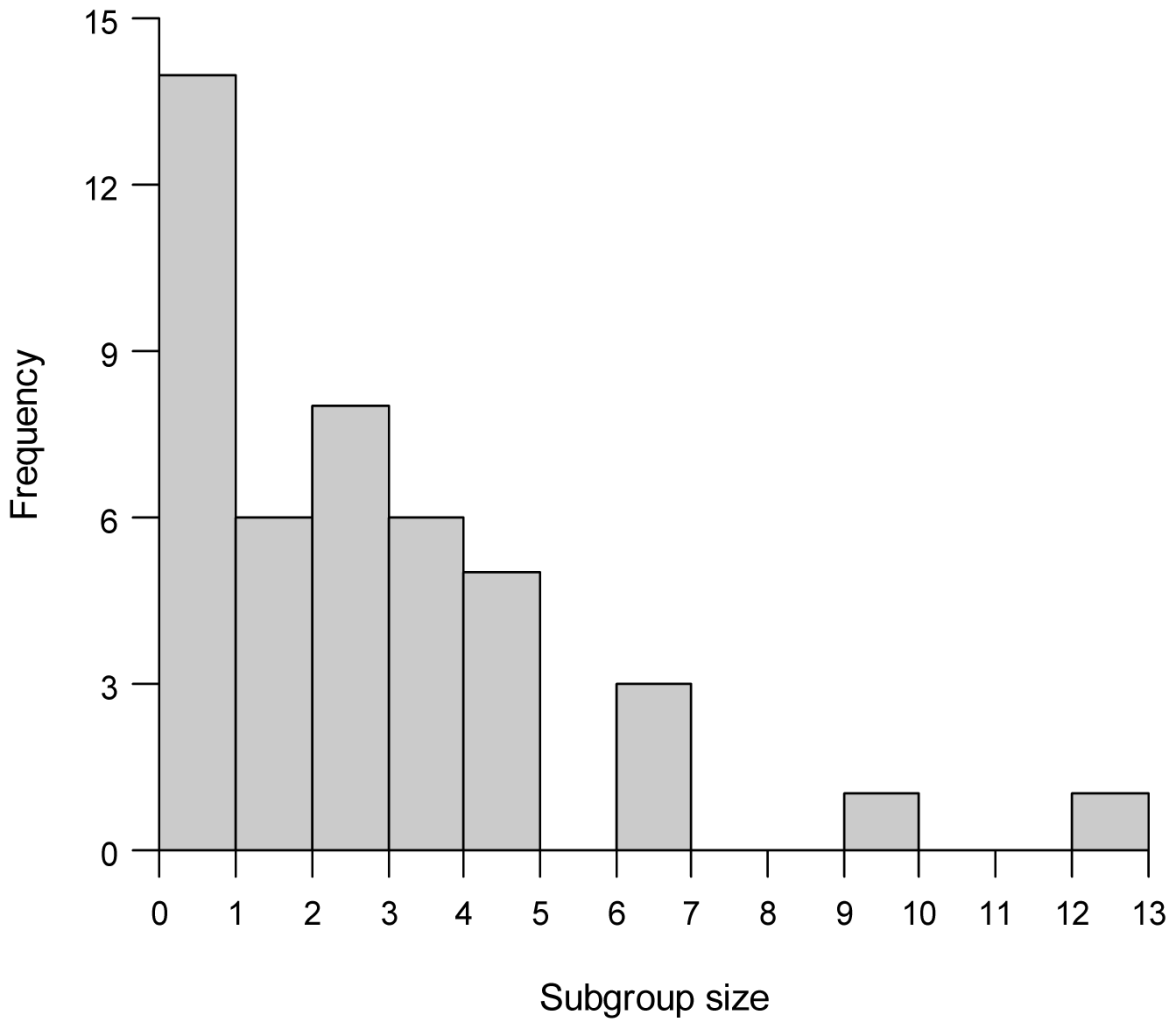
False killer whale subgroup sizes. Histogram of observed false killer whale subgroup sizes (n = 44) used in the estimation of expected subgroup size (E(s) = 3.11, CV = 0.12).

Forty-four values of observed false killer whale subgroup size were available for the estimation of *E*(*s*) (Fig. 4). These observations resulted from the group-size estimation protocol implemented during systematic-effort sightings McII 241 (*n* = 16 subgroups) and OES 86 (*n* = 18 subgroups) and off-effort sighting McII 61 (*n* = 6 subgroups). The aforementioned 2011 Palmyra EEZ survey and new passing mode protocol contributed four values of subgroup size, ranging from 1 to 7 individuals (PIFSC, unpublished data). The geometric mean of the “best” estimates of subgroup sizes was used in only seven cases, as generally only one observer provided a size estimate for a given subgroup. An average “best”:“low” size ratio (1.2; calculated from 22 subgroups) was used to establish subgroup size for 11 of the McII 241 subgroups for which “best” estimates were not provided. Bootstrap resampling of the assembled observations produced an *E*(*s*) of 3.11 (CV = 0.12) individuals.

The group-size estimation protocol was successfully implemented for one systematic-effort sighting in each stock, which required probabilistic determination of the expected number of detected subgroups. For the pelagic sighting McII 241, 10 of the 16 (62.5%) subgroups were within 4.5 km of the projected trackline (Fig. 2A), while seven of the 18 (38.9%) subgroups in the NWHI sighting OES 86 were within 4.5 km of the projected trackline (Fig. 2C). Thus, nine of the McII 241 subgroups and six of the OES 86 subgroups were subject to probabilistic selection according to the estimated probability of detection, which was computed using the point estimate of the half-normal scale parameter (σ = 1.88). The expected number of detected subgroups for sightings McII 241 and OES 86 is 6.2 and 4.4, respectively. The expected number of detected subgroups was also probabilistically determined for pelagic systematic-effort sighting McII 35, in which the group-size estimation protocol was unsuccessfully executed (Fig. 2B). Based on the total group size of this sighting (22.6; Table 1) and the estimate of *E*(*s*), the expected number of subgroups present in McII 35 is 7.3. Applying the average proportion of subgroups within 4.5 km of the projected trackline for sightings McII 241 and OES 86 (0.51), the expected number of McII 35 subgroups within 4.5 km is 3.7. Factoring in a 100% detection probability for the first subgroup and the average detection probability (0.51) for the remaining subgroups, the expected number of detected subgroups for McII 35 is 2.4. Given the expected number of detected subgroups for sightings McII 241 and McII 35 and the three other pelagic systematic-effort sightings of single subgroups, *n_pelagic_* is 11.6 subgroups. Because OES 86 was the only systematic-effort sighting of the NWHI stock, *n_NWHI_* is 4.4 subgroups. The division of on-effort survey coverage into 150-km effort segments for the bootstrap procedure resulted in 118 effort segments in the Hawaiian EEZ stratum and 26 in the NWHI stratum. Adjusting the effort distance to include the length of the projected on-effort trackline produced an *L_pelagic_* of 15,664 km and an *L_NWHI_* of 2,925 km. The bootstrap estimates of *n_i_*/*L_i_* for the pelagic and NWHI stocks are 0.07 (CV = 0.59) subgroups 100 km^−1^ and 0.15 (CV = 1.04) subgroups 100 km^−1^, respectively.

### Abundance Estimates

The 2010 abundance of pelagic stock false killer whales was estimated to be 1,552 (CV = 0.66; 95% CI = 479–5,030) individuals. The 2010 abundance of NWHI stock false killer whales was estimated to be 552 (CV = 1.09; 95% CI = 97–3,123) individuals. A summary of the stock-specific estimates of the line-transect parameters, density, and abundance is shown in Table 3.

**Table 3:** Estimates of line-transect parameters, density (individuals 100 km^−2^), and abundance for false killer whales in the pelagic region of the Hawaiian EEZ and the insular waters of the Northwestern Hawaiian Islands (NWHI) in 2010.

Stock	*f*(0)	*ESW*	CV	*E*(*s*)	CV	*n*/*L*	CV	*g*(0)	CV	Density	Abundance	CV	95% CI
Pelagic	0.43	2.31	0.11	3.11	0.12	0.07	0.59	0.76	0.14	0.07	1,552	0.66	479–5,030
NWHI	0.43	2.31	0.11	3.11	0.12	0.15	1.04	0.76	0.14	0.13	552	1.09	97–3,123

*f*(0) = the probability density function of the perpendicular detection distances evaluated at zero distance.

*ESW* = the inverse of *f*(0) and the distance (in km) from the trackline for which as many individuals were detected beyond as were missed within.

*E*(*s*) = the expected size of false killer whale subgroups.

*n*/*L* = the subgroup encounter rate (presented in subgroups 100 km^−1^).

*g*(0) = the probability of detection on the trackline.

The coefficient of variation (CV) is shown for all parameters, and the lognormal 95% confidence interval (CI) is included for the abundance estimates.

## Discussion

The group-size estimation protocol represented a substantial change in data collection methodology for false killer whales, which had to be accommodated in the abundance estimation. This protocol was established because previous studies had demonstrated that visual observers were not detecting all false killer whale subgroups in a group, with the result that overall group sizes were likely being underestimated [18]. As total group size has generally served as the unit of detection and analysis in cetacean studies [7], an emphasis on obtaining more accurate estimates of false killer whale group size appeared warranted. In hindsight, while the group-size estimation protocol provided an effective way to assess the size and spread of false killer whale groups, it produced data that were difficult to analyze using standard line-transect methods. However, the protocol sightings did reveal the extreme degree to which false killer whale groups do not adhere to the definition of cluster associated with line-transect methodology [6] and therefore should not serve as the analytical unit. In contrast, subgroups are more aligned with the cluster concept and represent what is first detected by a visual observer, not the group as a whole, which may extend far beyond viewing range. Thus, subgroups are a more appropriate analytical unit, an adjustment that was applied post-hoc in the present estimation, but should be more fully integrated into future data-collection protocols. In that regard, PIFSC has instituted a revised protocol for false killer whales, whereby the ship remains on the trackline in passing mode while the visual observers make subgroup detections until the group passes the beam of the ship.

The line-transect abundance estimation approach employed in the present analysis departed from that previously used for Hawai’i pelagic false killer whales [18], [21] and other cetaceans [22]. The multiple-covariate framework, which addresses heterogeneity in the detection function and thus accommodates the pooling of sightings from multiple surveys, was not used because that approach links the estimation of the other line-transect parameters to the sightings of the focal study. In the current analysis, this linkage would have limited the ability to adequately represent uncertainty in those parameters and would not have allowed for adjustments to address the variability introduced by the group-size estimation protocol during HICEAS 2010. Using the conventional form of the line-transect estimator and separately estimating each of the parameters offered a workable way to appropriately incorporate data obtained from various sources and collected with different methods and thus produce the most robust and unbiased abundance estimate possible.

As in the multiple-covariate approach, previous sightings were pooled with those from HICEAS 2010 to model the detection function of false killer whale subgroups. However, because the multiple-covariate approach was not used, heterogeneity from factors other than perpendicular distance had to be minimized in the pooled sample. To reduce the impact of other species on the detection process, sightings of other large delphinids and mixed-species false killer whale sightings were excluded from the pooled sample, in contrast to detection function models previously applied to false killer whales [18], [21]. Exploratory analyses determined that other sources of discernible heterogeneity were the effects of geographical region (eastern tropical or central North Pacific) and Beaufort sea state, which are likely correlated because of the rougher seas within the central North Pacific. When the sighting pool was refined to include only the higher sea state conditions (i.e., Beaufort states 3–6) more frequently encountered in the central North Pacific, this heterogeneity was no longer detected statistically. It is possible that heterogeneity from other factors remained in the pooled sample, but was not detectable with the available sample size. The point estimate of *ESW* (2.31 km) presented here does not differ appreciably from that previously attributed to Hawai’i pelagic false killer whales (2.24 km) [18] and other large delphinids [21], [22].

The histogram of subgroup detections by perpendicular distance indicated that they were seen disproportionately more often close to the trackline (Fig. 3), which is not ideal for modeling the detection function [6] and is suggestive of false killer whale movement toward the ship prior to detection by the visual observers. One of the primary assumptions of line-transect sampling is that objects are detected prior to a response toward or away from the observer [6]. Attractive movement leads to a reduced estimate of *ESW* and thus results in a density estimate that is positively biased. Vessel attraction has been documented for other cetacean species [28], [29], and there are anecdotal records of such behavior for false killer whales, both during research surveys (SWFSC and PIFSC, unpublished data) and by longline fishermen [30]. For 64.3% (*n* = 9) of the HICEAS 2010 false killer whale sightings, the visual observers noted on the sighting forms that animals were moving toward the ship. Additionally, acoustic observers during several surveys have recorded false killer whales in close proximity to the towed hydrophone array, both before and after detection by the visual observers (SWFSC and PIFSC, unpublished data). Targeted analysis of available acoustic data may yield important insights into the magnitude of vessel attraction by false killer whales at various distances from the trackline and allow for the estimation of correction factors aimed at reducing the positive bias in density estimates. At present, the use of the half-normal model to estimate the detection function minimized the impact of vessel attraction (Fig. 3), but could not entirely eliminate a positive bias of unknown magnitude in the abundance estimates.

The estimate of *E*(*s*) indicates that false killer whale subgroups are generally small, although there is variation (Fig. 4) that will likely be better characterized as more observations of subgroup size become available during future surveys. As this sample size increases, it will also be possible to examine the potential effect of subgroup size on the detection process. The recently revised false killer whale protocol will facilitate the detection of subgroups in a more analytically appropriate manner, although because the ship is required to remain in passing mode and not divert from the trackline for subgroup size assessment, the protocol might introduce greater uncertainty in estimates of subgroup size [31]. However, the revised protocol represents a logistically feasible tradeoff to obtain estimates of subgroup size and encounter rate that are consistent with line-transect assumptions.

For the present estimation of subgroup encounter rate, an approach was developed that incorporated the subgroups associated with sightings for which the group-size estimation protocol was attempted (Fig. 2). Although it is unknown how many subgroups would have been visually detected had the ship remained on the trackline, counting each of these sightings as a single detection would have led to the underestimation of encounter rate. The approach employed probabilistic sampling of subgroups according to their distance from the projected trackline and the estimated detection function. However, these subgroups were localized as the ship was moving toward them, such that their detection location may not represent their original position with respect to the projected trackline. This possibility introduces a potential source of bias of unknown magnitude and direction into the estimation process. The probabilistic sampling approach was expanded to deal with sighting McII 35, for which the group-size estimation protocol was not successfully completed (Fig. 2B). Neglecting to factor in the known presence of multiple subgroups for this sighting would have underestimated the encounter rate (0.05 subgroups 100 km^−1^ instead of the present estimate of 0.07 subgroups 100 km^−1^) and led to an underestimate of abundance. For this reason, information from other sightings and parameters was used to estimate the expected number of detected subgroups for this sighting, reducing potential downward bias in the estimation, although introducing additional uncertainty.

The estimated line-transect parameters resulted in density estimates of 0.07 (CV = 0.66) individuals 100 km^−2^ for the pelagic stock and 0.13 (CV = 1.09) individuals 100 km^−2^ for the NWHI stock (Table 3). Cetacean density in the Hawaiian EEZ is known to be lower than most other surveyed areas [21]. However, the density of pelagic false killer whales as estimated in 2010 and 2002 (0.02 individuals 100 km^−2^, CV = 0.93) [18] is among the lowest estimated for each species that occurs year-round in the study area [21]. Little is known about how false killer whales use the pelagic environment of the Hawaiian EEZ, making it difficult to make inference about density. Characterizing the oceanographic environment associated with false killer whale detections would be informative in this regard and could be used to parameterize habitat-based density models [32]. The 2010 density of false killer whales in the insular waters of the NHWI, although also comparatively low [21], was estimated to be two times that of pelagic stock. While this higher density could be related to increased productivity around the Hawaiian Islands [33], the density of the main Hawaiian Islands stock (0.05 individuals 100 km^−2^, determined by dividing the reported abundance estimate by the stock area) [12] is more similar in magnitude to the pelagic stock. However, false killer whales in the main Hawaiian Islands have experienced a population decline, indicating that this region can likely support a higher density of false killer whales. Further, the density estimate for the NWHI stock is imprecise and may be positively biased due to the effect of insular-type false killer whale social structure. False killer whales in the main Hawaiian Islands have been determined to belong to a single social network [9], [34]. The coarse-scale spatial coverage of the NWHI during HICEAS 2010 compounded by this social structure could result in overestimates of density if the stock tends to occur as a few large, closely-associated groups. Mark-recapture techniques may prove a more suitable means by which to estimate the abundance of the NWHI stock of false killer whales, as is the case for the main Hawaiian Islands stock [12].

The abundances of the Hawai’i pelagic and NWHI false killer whale stocks were estimated to be 1,552 (CV = 0.66; 95% CI = 479–5,030) and 552 (CV = 1.09; 97–3,123) individuals, respectively. The greater density of pelagic false killer whales in 2010 translated into a higher abundance estimate than the 484 (CV = 0.93; 95% CI = 103–2,274) individuals estimated to be in the study area in 2002 [18]. The log-normal 95% CI of the 2002 and 2010 pelagic stock estimates overlap, suggesting that the estimates are not significantly different between the two years, although computing the confidence interval for the difference of population means is a more robust and appropriate comparison [35]. However, the estimates are based on shared data that were used to model the detection function associated with each estimate. Thus, the 2002 and 2010 estimates of pelagic false killer whale abundance are not statistically independent and cannot be compared in standard statistical tests. While the abundance estimate of false killer whales in the NWHI is the best available, it is a function of the area used for the stock range, which is presently uncertain, particularly in its western extent (Fig. 1). A better elucidation of this range through telemetry and photo-identification studies is recommended and would likely result in adjustments to the abundance estimate. As previously mentioned, both estimates are presumably positively biased as a result of false killer whale vessel attraction, although the extent of the bias is unknown. Until this phenomenon can be better quantified by acoustic analysis or potentially independent observer studies [36], relevant correction factors are unavailable.

The highly variable nature of false killer whale behavior and grouping patterns creates the potential for a number of biases and uncertainties in line-transect abundance estimation. This effect is compounded by the inevitable logistical and technical difficulties that arise when operating in remote areas at sea. The present analysis attempted to address these characteristics, minimize bias, and quantify uncertainty as appropriately as possible. Explicitly accounting for subgroup structure was determined to be the best approach for analyzing the HICEAS 2010 false killer whale detections and is recommended for future false killer whale line-transect data collection and abundance estimation. Using subgroups as the detection and analysis unit is also likely applicable to other cetaceans with multilevel social organization, such as pilot whales (*Globicephala* spp.) [37], killer whales (*Orcinus orca*) [38], and sperm whales (*Physeter macrocephalus*) [39], particularly when associated subgroups are separated by large distances. This approach could also be considered for non-cetacean populations with subgroup structure. For example, primates are widely surveyed using line-transect methods, but the effects of group size, spread, and response are known to bias estimates of abundance [40]. Subgroup-based detection has been suggested for primate line-transect surveys because it is no longer necessary to detect every subgroup in a group aside from those on or near the trackline [23]. While characterizing groups in their entirety is important for understanding the ecology and social dynamics of a population, it may need to be decoupled from the line-transect survey process in order to produce more reliable estimates of abundance.
